# Transcatheter and Surgical Approaches to Addressing the Complications of Transcatheter Procedures: Current Trends and Future Challenges

**DOI:** 10.3390/jcdd13030143

**Published:** 2026-03-19

**Authors:** Antonio Nenna, Giovanni Casali, Giuseppe Patti, Carmelo Dominici

**Affiliations:** 1 Department of Cardio-Thoracic-Vascular Diseases, Foundation IRCCS Ca’ Granda Ospedale Maggiore Policlinico, 20122 Milan, Italy; carmelo.dominici@policlinico.mi.it; 2 Department of Cardio-Thoracic-Vascular Diseases, Ospedale Maggiore Della Carità, 28100 Novara, Italy; giovanni.casali@maggioreosp.novara.it (G.C.); giuseppe.patti@uniupo.it (G.P.)

Transcatheter structural heart procedures, including transcatheter aortic valve replacement (TAVR) and transcatheter edge-to-edge repair (TEER), have revolutionized the management of valvular heart diseases in selected or high-risk patients. By offering non-surgical alternatives to traditional open-heart surgery, these techniques have significantly improved clinical outcomes, especially for patients deemed to be at high or prohibitive surgical risk. However, despite rapid technological advancements, evolving device iterations, and increasing operator experience, percutaneous interventions still carry a residual risk of severe complications. The overall number of procedures is rising in every country, and a parallel trend can be observed for complications; therefore, an adequate understanding of these adverse events, along with their prompt treatment, is crucial for optimizing patient safety.

A major concern following TAVR is the development of infective endocarditis (IE). Despite technological advances, its incidence remains relatively stable, and its cumulative risk (over time) presents unique diagnostic and therapeutic challenges due to its complex microbiological profile, atypical clinical presentations, and persistently high mortality rates related to both the natural history of the disease and surgical explant of infected prostheses [1]. Besides infectious complications, structural and mechanical emergencies during or immediately after TAVR can also be catastrophic. Major intraprocedural complications—such as annular ruptures, severe paravalvular regurgitation, and coronary obstructions—frequently necessitate emergent cardiac surgery [2,3]. Among the most devastating of these structural failures is acute aortic dissection following TAVR [4,5]. Although considered a rare occurrence, iatrogenic aortic dissection is a frequently lethal intraprocedural sequela that requires immediate recognition, typically via advanced imaging, and rapid surgical conversion to prevent a profound hemodynamic collapse.

The landscape of transcatheter interventions has also rapidly expanded to the treatment of atrioventricular valve pathologies. Transcatheter strategies for mitral and tricuspid regurgitation, primarily using TEER, have demonstrated excellent efficacy and safety profiles [6,7]. Nevertheless, these procedures are not immune to serious intraprocedural complications. Unforeseen mechanical emergencies, such as ascending aortic dissection during TEER, have been documented, highlighting the fragility of cardiovascular structures during complex catheter navigation [8]. Furthermore, transcatheter-device-related closure of mitral paravalvular leaks carries its own set of technical challenges and risks, such as device embolization and interference with adjacent valvular structures, resulting in acute massive valve dysfunction [9]. The incidence and occurrence described in “classic” published papers might differ from the “real world scenario” owing to “publication bias”.

In conclusion, while transcatheter structural heart procedures serve as life-saving alternatives for a broad spectrum of patients, they carry an inherent risk of devastating complications. Rigorous pre-procedural anatomical imaging, ongoing refinement of percutaneous techniques, and the constant availability of emergent surgical (and, above all, skilled) backup remain paramount for safely managing these complex cardiovascular interventions. This Special Issue summarizes the latest advances in the treatment of transcatheter-procedure-related complications. Of the contributions received, seven highly relevant manuscripts have been accepted for publication. The topics encompass a wide range of procedures and complications, shedding light on a new integrated approach that allows the Heart Team to demonstrate its quality. Based on this Special Issue, we are confident that cardiologists and cardiac surgeons participating in modern Heart Teams can easily improve their knowledge by learning about the latest trends in “how to treat complications of transcatheter procedures,” as presented by experts in the field.

## 1. Hameau et al. (Contribution 1, Review): Management of TAVI Underexpansion with Self-Expanding Valves

TAVI involves the use of self-expanding valves that occasionally present the critical complication of underexpansion, which significantly compromises long-term clinical outcomes by increasing the risk of paravalvular leaks, valve thrombosis, and overall mortality. This structural failure is primarily driven by three distinct mechanisms: prosthesis infolding, incorrect guidewire crossing sites, and true mechanical underexpansion caused by severe calcification. Understanding the specific pathophysiology behind these mechanisms is essential for developing effective preventative and corrective strategies. Comprehensive pre-procedural planning, combined with targeted intraoperative techniques, is required to ensure adequate valve deployment and mitigate these adverse events, thereby improving both immediate and long-term patient prognoses.

## 2. Liscano et al. (Contribution 2, Systematic Review): A Meta-Learning Model to Predict Cohort-Level Mortality After TAVR

The extreme heterogeneity in mortality outcomes following TAVR presents a significant challenge that conventional meta-analyses often fail to elucidate. To address this limitation, Liscano et al. developed an innovative meta-learning model utilizing aggregate cohort-level data from 58 studies encompassing over 533,000 patients. While traditional models demonstrated extreme unexplained variance, the advanced optimized machine learning algorithm successfully explained 65.3% of the variability in mortality. Interpretability analyses identified the Society of Thoracic Surgeons Predicted Risk of Mortality (STS-PROM) score, recruitment year, the percentage of transfemoral approaches, and diabetes prevalence as the primary predictors. This approach demonstrates that meta-learning can successfully transform seemingly unexplained clinical heterogeneity into highly interpretable, prognostic patterns.

## 3. De Gregorio et al. (Contribution 3, Review): Antithrombotic Therapy in TAVI: Focus on Gender Differences

Optimizing antithrombotic therapy following TAVI requires maintaining a delicate balance between mitigating thromboembolic risks, such as stroke or valve thrombosis, and preventing major bleeding events. This balance is particularly complex in regard to women, who exhibit distinct anatomical and physiological characteristics—including smaller vessel calibers, increased vascular tortuosity, and altered platelet reactivity—that uniquely influence their clinical risk profiles. Consequently, female patients experience higher rates of major bleeding and vascular complications post-TAVI, despite demonstrating survival outcomes comparable or superior to those of men. These gender-based discrepancies highlight the critical need for sex-specific risk stratification and tailored antithrombotic regimens to improve safety and long-term management in this expanding patient demographic.

## 4. Ferraresi et al. (Contribution 4, Review): Left Ventricular Apical Cannulation in Acute Type A Aortic Dissection

In the surgical management of acute iatrogenic (i.e., during TAVI) type A aortic dissection, the selection of an arterial cannulation site fundamentally dictates early hemodynamics, organ protection, and the incidence of malperfusion. Transapical left-ventricular cannulation (TAC) has emerged as a highly effective “central” strategy for rapidly initiating cardiopulmonary bypass (CPB) while ensuring antegrade true-lumen flow. This technique, which involves advancing a long arterial cannula through the left-ventricular apex and across the aortic valve under echocardiographic guidance, is particularly advantageous for hemodynamically unstable patients or those with unsuitable peripheral vessels. TAC significantly reduces skin-to-pump times and yields early mortality and stroke rates comparable to those for traditional approaches, although careful execution is required to prevent localized complications like apical bleeding.

## 5. Moroz et al. (Contribution 5, Original Article): Clinical Significance of TAPSE/PASP Ratio in Risk Stratification for TAVR

Aortic stenosis progressively increases left-ventricular afterload, ultimately precipitating ventricular dysfunction and heart failure. In patients undergoing TAVR, evaluating right-ventricular adaptation to pressure overload via the tricuspid annular plane systolic excursion to pulmonary systolic arterial pressure (TAPSE/PASP) ratio offers critical prognostic value. A retrospective analysis of 100 TAVR patients revealed that a lower median TAPSE/PASP ratio correlates significantly with adverse clinical outcomes. Specifically, lower ratios were associated with a higher incidence of post-procedural atrial fibrillation, a decreased left-ventricular ejection fraction, prolonged hospitalization, and heightened systemic inflammatory responses. Consequently, the TAPSE/PASP ratio serves as a valuable, non-invasive risk-stratification marker with which to identify high-risk individuals and optimize peri-procedural management.

## 6. Compagnone et al. (Contribution 6): Transfemoral TAVI at Hospitals Without On-Site Cardiac Surgery (Study Protocol)

While current international guidelines stipulate that transcatheter aortic valve implantation (TAVI) should strictly be performed in facilities equipped to conduct on-site cardiac surgery (CS), the rapid evolution of valve technology and operator expertise has drastically reduced procedural complications. Emergency rescue surgery is currently required in a few cases, and the outcomes of these catastrophic events remain uniformly poor even with on-site surgical support. The “TAVI at Home” protocol outlines a multicenter, prospective interventional study intended to evaluate the safety and feasibility of performing TAVI in centers lacking on-site CS. By employing rigorous Heart Team evaluations and requiring high-volume interventional expertise, this decentralized approach is designed to safely expand procedural access, reduce critical waiting times, and ensure continuity of care for fragile patients. This is just a study protocol, and results are awaited.

## 7. Synetos et al. (Contribution 7): Paravalvular Leak in TAVI: Current Challenges and Future Directions (Review)

Transcatheter aortic valve implantation (TAVI) has revolutionized the therapeutic landscape for severe aortic stenosis, successfully expanding its indications from prohibitive-risk patients to intermediate- and low-risk populations. However, as the procedure extends to younger individuals with longer life expectancies, mitigating complications such as paravalvular leaks (PVLs) is becoming increasingly paramount. A PVL occurs when regurgitant blood flows through structural gaps between the prosthetic device and the native annulus, leading to left-ventricular remodeling and reduced cardiac efficiency. This comprehensive review evaluates the incidence, anatomical and device-specific predictors, and clinical implications of PVLs across various TAVI platforms. By highlighting advanced pre-procedural imaging and novel transcatheter closure strategies, the study emphasizes the critical need to eliminate PVLs to optimize long-term clinical outcomes.
